# Postoperative Deep Sedation after Microvascular Reconstructive Surgery for Oral Cancer Increases the Risk of Early Postoperative Pneumonia

**DOI:** 10.3390/dj11050137

**Published:** 2023-05-18

**Authors:** Satoshi Fukuzawa, Kenji Yamagata, Shohei Takaoka, Fumihiko Uchida, Naomi Ishibashi-Kanno, Toru Yanagawa, Hiroki Bukawa

**Affiliations:** Department of Oral and Maxillofacial Surgery, Institute of Clinical Medicine, Faculty of Medicine, University of Tsukuba, 1-1-1 Tennodai, Tsukuba 305-8575, Ibaraki, Japan; y-kenji@md.tsukuba.ac.jp (K.Y.); sho.tmdu@gmail.com (S.T.); uchiyamada1031@yahoo.co.jp (F.U.); greened_amethyst829@hotmail.com (N.I.-K.); ytony@md.tsukuba.ac.jp (T.Y.); hiroki.bukawa@gmail.com (H.B.)

**Keywords:** oral cancer, postoperative delirium, postoperative pneumonia, postoperative sedation, reconstructive surgery

## Abstract

This study investigated the effect of postoperative deep sedation after oral cancer reconstructive surgery on the occurrence of early postoperative pneumonia and early postoperative delirium. We obtained medical records of 108 consecutive patients who underwent microvascular reconstructive surgery at Tsukuba University Hospital for oral cancer between January 2013 and December 2021. Forty-six of them woke soon after surgery. Ten of these forty-six patients were restless and required immediate sedation within 3 h after surgery. The comparison between sedation group and no sedation group revealed early postoperative pneumonia in the no sedation group; however, sedation was not related to early postoperative delirium. The preoperative albumin levels of patients with postoperative pneumonia were significantly different (*p* = 0.03) than those of patients without postoperative pneumonia. The performance status (*p* = 0.02), preoperative albumin level (*p* = 0.02), and age 75 years or older (*p* = 0.02) were significantly associated with postoperative delirium. Restless patients and those who could not be sedated experienced delirium and pneumonia. The risk of pneumonia was increased for patients who were difficult to sedate.

## 1. Introduction

Oral cancer is associated with a high mortality rate; in 2018, there were approximately 355,000 new cases of oral cancer worldwide, and 177,000 individuals died as result [1]. There are several treatments for oral cancer currently. The advent of immune checkpoint inhibitors has been reported to prolong the life expectancy, but only in recurrent and metastatic cases. Hence, the mortality rate is still high [2].

Regarding the treatment of oral cancer, if surgery is possible, then it is the first choice. If the resection area is large, then it may be accompanied by dysfunctions in speech, eating, and swallowing, as well as aesthetic disorders; therefore, reconstructive surgery is often performed at the same time as cancer treatment surgery. Engraftment of free flaps after oral cancer resection and reconstruction is essential for the recovery of postoperative oral function. The flap engraftment rate is 96% to 99% [3]; however, there is a risk of free flap necrosis when the anastomotic vessel is obstructed because of thrombosis or hematoma compression. Cornejo et al. conducted a retrospective observational study, involving 170 postoperative patients, including trauma and head and neck cancer patients, who underwent free flap transplantation, in a location where complications are likely to occur within 72 h postoperatively and were admitted to the intensive care unit (ICU) for monitoring. Flaps should be evaluated every 1 to 2 h [4]. Therefore, extension, compression, and neck rotation are prohibited because of postoperative flap engraftment. All patients must be kept awake because the oral cavity requires evaluation every 1 to 2 h. Therefore, their environment promotes agitation and delirium. Postoperative sedation management, respiratory and circulatory management, and flap management are commonly performed during flap management. However, the complications of sedation include atelectasis and postoperative pneumonia. This study retrospectively examined the effects of sedation and awake management on the occurrence of early post operative pneumonia (EPOP) and early postoperative delirium (EPOD) that developed postoperatively.

## 2. Materials and Methods

### 2.1. Study Design and Sample

This study primarily included patients diagnosed with oral cancer from January 2013 to December 2021 at the Department of Dental and Oral Surgery, Tsukuba University Hospital (Ibaraki, Japan). Patients underwent reconstructive surgery with free flaps. The records of 108 consecutive patients who underwent major head and neck tumor resection and reconstructive surgery and received postoperative management in the high care unit (HCU) or ICU were analyzed retrospectively. Reconstructive surgery was not performed for patients with unresectable cancer and a poor general condition. The primary resection and neck dissection for oral cancer were performed by an oral surgeon, and the free flap procedure was performed by a plastic surgeon. All patients underwent tracheostomy. Forty-six patients who underwent surgery from March 2015 to September 2019 were awakened while in the operating room. 

### 2.2. Definition of the Patient Group

We defined patients who were sedated after surgery as the sedation group (SG). We defined patients who woke after surgery as the trying to awaken group (TAG). We defined patients who were unable to sufficiently rest and required sedation again as the re-sedation group (RSG). We defined patients who did not require sedation as the no sedation group (NSG).

### 2.3. Patient Management

Dexmethidine was used for both the SG and TAG patients to sustain postoperative rest. The SG was managed with deep sedation using propofol, and respiratory depression was managed with a respirator. TAG patients who developed symptoms such as excitement and delirium were not able to sufficiently rest and received sedative management in the HCU, ICU, or operating room and were further defined as the RSG. All cases were targeted to maintain a Richmond Agitation Sedation Scale (RASS) score of 0 to 2 until the postoperative period.

When the blood pressure of the patient decreased significantly because of sedation, dopamine hydrochloride was administered to increase and maintain the systolic blood pressure at 100 mmHg or more. The morning after surgery, sedation was weaned. If the patient was in a state of rest, then the ventilator was withdrawn, and sedation was released. When entering the HCU or ICU, the airway of the patient was assumed to be the tracheostomy. For all patients who received preoperative oral care, specialized oral care was performed within 5 days of surgery. Postoperative oral care involved cleaning, with a sponge brush or cotton ball.

### 2.4. Patient Exclusion Criteria

The study excluded patients who underwent repeat surgery within 1 week postoperatively because of problems such as poor flap engraftment and congestion, patients using benzodiazepine, histamine-2 blocker, or anticholinergic drugs postoperatively, and patients with dementia. Additionally, patients with a history of alcoholism, stimulant use, or psychiatric diseases were excluded. Midazolam is associated with a higher risk of delirium than other anesthetics [5]; however, none of the patients received midazolam.

### 2.5. Definitions of EPOP and EPOD

The primary endpoints were EPOP and EPOD. EPOP was defined as the occurrence of postoperative pneumonia with a fever of 37.5 °C or higher, increased C-reactive protein level, abnormal chest radiography findings, sputum, and no local infection within 1 week postoperatively. Pneumonia occurring within 7 days of admission to the ICU was also classified as EPOP in this study. Agitation immediately after surgery was not classified as EPOD. In this study, only short-term delirium was evaluated because other factors significantly affect delirium during hospitalization, and it may not be related to postoperative sedation. This differed from the definition of delirium to evaluate the effect of postoperative sedation during this study.

Delirium attributable to long-term hospitalization often is not considered related to postoperative sedation. This study focused on short-term delirium, and the evaluation was used as the endpoint. The Diagnostic and Statistical Manual of Mental Disorders, 5th edition, diagnostic criteria for delirium were used [6].

### 2.6. Pain Control and Assessment

Postoperative pain was evaluated using the numerical rating scale (NRS) for patients, who were not sedated 48 h postoperatively. Acetaminophen 60 mg/kg/day was administered to provide analgesia, and nonsteroidal anti-inflammatory drugs were also administered as needed.

### 2.7. Statistical Analysis

A statistical analysis was performed using JMP Pro (SAS Institute Inc., Cary, NC, USA). *p* < 0.05 was considered statistically significant.

Continuous variables were examined using Welch’s *t*-test. Nominal variables were examined using Fisher’s exact test. Based on the results obtained, a forced-entry multivariate logistics regression analysis of the significance factor and sedation factor was performed.

## 3. Results

### 3.1. Patient Characteristics

**Figure 1:**
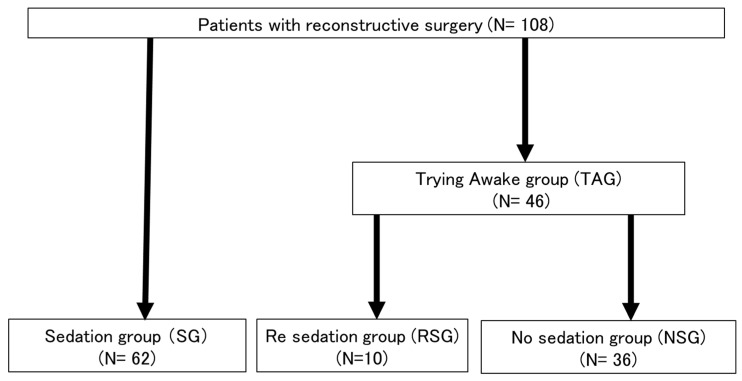
Flow chart of patient groups.

There were 62 patients in the SG and 46 patients in the NSG. The TAG patients, who were awakened and then sedated again without resting, were further classified as the RSG. The remaining patients in the TAG were classified as the NSG (Figure 1). The background characteristics of the patients in both groups were examined. A significant difference in the performance status (PS) was observed between the two groups, but no other significant differences were observed (Table 1). All patients except for three were managed by inhalation anesthesia during surgery. There are two methods used for inhalation anesthesia management propofol for sevoflurane management and desflurane for maintenance of anesthesia during surgery. The three aforementioned patients were managed with total intravenous anesthesia (TIVA). Postoperative complications, specifically EPOP and EPOD, of patients in the SG and NSG were evaluated. There was a significant difference in EPOP (*p* = 0.02).

### 3.2. Effect of Sedation

The sedation effect was evaluated in all groups, except for the RSG (Table 2). SG and NSG had significantly different PS (*p* = 0.02), sevoflurane (*p* = 0.02), and postoperative NRS (*p* = 0.02). There were no associated factors with PS. Sevoflurane, which results in a slower awakening time, was more commonly selected because it did not require the anesthesiologist to keep the patient awake. The NRS scores of both groups were significantly different. It was possible to relieve sleep disorders and pain immediately postoperatively using sedation as a management strategy.

### 3.3. Effect of Agitation

A univariate regression analysis of the RSG and NSG was performed (Table 3). The body mass index (BMI) (*p* = 0.03) and hypertension status of patients were significantly different, but no other significant differences were observed. EPOD was significantly observed in the RSG. However, agitation had no effect on EPOP.

We examined both the SG and the RSG, and we observed EPOP and EPOD in the RSG (Table 4).

### 3.4. Examination of EPOP

A univariate regression analysis of EPOP showed a significant difference in preoperative albumin levels of patients with and without EPOP (Table 5). However, there was no significant difference in sedation or the sedation period. There was a significant difference in sedation or the sedation period of patients who developed EPOD. This was partly attributable to the increased risk of delirium for patients with pneumonia. A multivariate analysis of the preoperative albumin levels and sedation was performed (Table 6), and a significant difference in the preoperative albumin levels of patients with and without EPOP was observed.

### 3.5. Examination of EPOD

A univariate regression analysis of delirium showed significant differences between the PS (*p* = 0.02), preoperative albumin level (*p* = 0.02), and age (age 75 years or older) of patients (*p* = 0.02) with and without EPOD (Table 7). No significant difference in sedation was observed between patients with and without EPOD. The sedation period was slightly longer in patients with EPOD, likely because of their inability to sufficiently rest.

## 4. Discussion

Treatment methods such as cell repair are being researched as alternatives to reconstructive surgery for oral cancer, but a long period of time is required before they can be applied to patients [7]. Many treatment methods have been developed, including immunotherapy and boron neutron capture therapy [8,9,10]; however, surgical treatment remains the first option, and reconstructive surgery remains necessary. Management methods involving fewer complications and less burden on the patient after reconstructive surgery are necessary.

This study found that EPOP and EPOD are significantly related. However, it was found that EPOP and EPOD were likely to occur in patients who were sedated because they were unable to sufficiently rest. The incidence of delirium after oral cancer reconstruction surgery is high; therefore, several research reports have been published. The incidence of postoperative delirium with head and neck surgery was 19.3% (11.5–36.1%) according to a meta-analysis by Yun et al. [11]. During our study, it occurred in 29 of 108 patients (26.8%). According to the results of our study, old age (70 years or older), male sex, duration of surgery, history of hypertension, blood transfusions, tracheotomy, American Society for Anesthesiologists (ASA) fitness grade of III or more, flap reconstruction, and neck dissection were related to a higher risk of delirium. There was a high incidence of delirium among patients who underwent surgery of the head and neck region. However, this was likely because of the high risk of flap reconstruction, tracheostomy, and repeat surgery associated with surgery in this region. Patients 75 years of age or older also were at higher risk for delirium. Shah et al. reported that surgery lasting more than 6 h places patients at risk for delirium [12]. Weed et al. reported that factors associated with delirium were old age (age 70 years or older), alcohol dependence, cognitive impairment, dysfunction, electrolyte imbalance, and glycemic disorders [13]. Because their study mainly involved postoperative sedation, patients with alcohol dependence or cognitive impairment were excluded. Additionally, all patients underwent tracheotomy with an average operative time of 10 h or more. Their study was conducted among a high-risk verification group. Preoperative hypoalbuminemia was also identified as a risk factor for postoperative delirium, and the results of our present study supported this finding [14,15]. Additionally, delirium was more common among patients for whom waking management was attempted but not maintained. Waking management was likely not feasible because patients with delirium required sedation. Patients cannot be kept awake without sevoflurane. However, one patient received TIVA with propofol and remifentanil, and the remaining patients were managed with desflurane. A retrospective study of elderly Japanese patients also reported no significant difference in the incidence of postoperative delirium with the use of sevoflurane and desflurane [16]. This finding was also observed in our study. Desflurane was more commonly used in patients who were not kept awake. Desflurane has a smaller blood/gas partition coefficient and a faster waking time [17]. Although the number of patients who cannot be kept awake is small, it is essential to identify these patients. Yang et al. conducted a prospective, double-blind, randomized controlled trial of 80 patients admitted to the ICU after free flap transplantation with microsurgery to determine factors associated with the prevention of agitation and delirium [18]. Patients who received a continuous infusion of dexmedetomidine were significantly more relaxed at the time of admission to the post-anesthesia care unit than patients in the placebo group (10.3% vs. 30.0%; *p* = 0.029). However, there was no significant difference between these two groups in terms of delirium (5.1% vs. 12.5%, *p* = 0.432) [18]. Based on these results, a continuous intravenous infusion of dexmedetomidine was deemed effective. In the present study, patients were sedated using dexmedetomidine, and propofol was used when needed. Dexmedetomidine was not used for patients who underwent trial awake management. This may have significantly influenced the outcomes of this study.

The incidence of postoperative pneumonia has been reported as 7.1% to 40% [12,19,20,21]. Furthermore, postoperative pneumonia reportedly occurs in 9.1% to 11.6% of patients who undergo surgery of the head and neck region surgery [19,22]. However, a postoperative pneumonia rate of 30.6% has been reported for patients with head and neck cancer who underwent reconstructive surgery [23]. The incidence of postoperative pneumonia in this study was 29.6% (32 of 108), which is similar to the reported rates. Li et al. reported that the risk factors for pneumonia among 482 patients who underwent oral cancer surgery with tracheotomy were being male, having a long tracheotomy procedure, and being smokers [24]. According to previous meta-analyses, smoking was a predictor of pulmonary complications [25,26]. However, a history of smoking was not identified as a risk factor for pneumonia by this study. Petrar et al. reported that older age and hypertension were strongly correlated with pulmonary complications [27]. However, no correlation was found during this study. The high incidence of postoperative pneumonia in this study was related to the decline in the swallowing function associated with the wide resected area that required reconstructive surgery. To minimize the risk of airway stenosis, tracheostomy was performed for all patients, and they were managed with a cuffed cannula. However, dripping occurred; this was likely because of the worsening swallowing function. Sedation was performed to protect the reconstructed flap to achieve a target of RASS score of 0 to 2. Patients with decreased blood pressure were managed with combined pressor agents.

Ventilator management was performed for the purpose of respiratory management with sedation. Respiratory depression was induced with propofol. According to a study of ventilator withdrawal, patients with withdrawal in the ICU had more medical complications (55.5% vs. 12.7%; *p* < 0.001) and a longer ICU stay than those with withdrawal in the operating room. (4.4 days vs. 2.9 days, *p* < 0.001) [28]. Prolonged ventilator management increases the risk of aspiration pneumonia and atelectasis. However, as a precondition, respiratory management was short-term; it lasted only until the day after surgery. Therefore, sedation did not affect the occurrence of pneumonia.

For patients who underwent reconstructive surgery, pharyngeal stenosis was caused by flap edema during entrance to the oral cavity, and the swallowing function was impaired. Therefore, the risk of postoperative pneumonia is high among patients who undergo reconstructive surgery for oral cancer.

This study initially predicted that postoperative sedation suppressed delirium and increased the risk of pneumonia. However, the results were different. Agitated patients were sedated again, and they tended to be more difficult to sedate. Pneumonia has been associated with numerous complications. The occurrence of delirium made oral care difficult or inadequate.

Although EPOP and EPOD were not affected, even without sedation management, patients experienced great pain immediately after surgery when the flap was evaluated. Therefore, postoperative sedation management is preferred. However, if sedation significantly lowers the blood pressure and hemodynamic management becomes challenging, then postoperative sedation is not performed, or management with shallow sedation is considered. Initially, sedation was deemed to have no effect on the occurrence of delirium. There was no significant difference in the sedation period because only short-term sedation was performed. Sedation was ended as soon as possible for all patients. Based on a systematic review, there were no significant differences in flap survival, reoperation, readmission, respiratory failure, delirium, and mortality; moreover, none of the patients required postoperative transfer to the ICU or HCU [29]. Therefore, postoperative sedation is not always necessary. The results of this study showed that the difference between sedated and awake patients was insignificant, and neither management option was superior. However, EPOP and EPOD occurred in patients who did not tolerate awake management. For patients who are unable to tolerate postoperative waking management, postoperative sedation is preferred. Additionally, the ICU response, environment, and circumstances at each facility are different. Not all patients can tolerate awake management; therefore, their management should be individualized accordingly. After patients awaken, it becomes clear who will and will not require repeat sedation. Management should be performed appropriately based on the needs of the individuals.

### Limitations

EPOP and EPOD are influenced by several factors. The number of cases in this study was too small to allow identification factors associated with the inability to maintain postoperative wakefulness. Further studies, involving patients with and without ICU management, should be conducted to confirm the findings of this study.

## 5. Conclusions

Many EPOP and EPOD cases were observed in the RSG. It is desirable to manage such groups with initial sedation; however, this study did not clarify which types of risk corresponded to this RSG. Therefore, it is necessary to elucidate these risk factors in the future.

## Figures and Tables

**Table 1:** Patient characteristics (* *p* < 0.05).

Variable	SG (N = 62)	TAG (N = 46)	*p*-Value
**Patient background**			
Age, Ave ± SD	66.3 ± 11.0	66.0 ± 10.8	0.89
Age, over 75 years (cases, %)	12 (19.4)	12 (26.1)	0.41
Gender, male:female	42:20	31:15	0.97
BMI, Ave ± SD	22.2 ± 2.96	22.2 ± 3.1	0.38
Perfomance Status (0–4), Ave ± SD	0.03 ± 0.17	0.17 ± 0.38	0.02 *
ASA, Ave ± SD	2.34 ± 0.65	2.37 ± 0.53	0.79
Hypertension (cases, %)	31 (50.0)	22 (47.8)	0.82
**The location of cancer**			0.36
Tongue (cases, %)	31 (50.0)	19 (41.3)	
Buccal (cases, %)	5 (8.0)	6 (13)	
Oral floor (cases, %)	3 (4.8)	4 (8.7)	
Mandibular (cases, %)	22 (35.5)	16 (34.8)	
Maxillar (cases, %)	1 (1.6)	0 (0)	
**Preoperative condition**			
Preoperative Alb (g/dL), Ave ± SD	3.94 ± 0.42	3.95 ± 0.45	0.98
Preoperative Cre (mg/dL), Ave ± SD	0.91 ± 1.28	0.75 ± 0.22	0.31
Preoperative Hb (g/dL), Ave ± SD	12.8 ± 1.99	13.2 ± 0.27	0.24
**Anesthesia method**			0.31
Inhalation anesthesia (cases, %)	61 (98.4)	43 (93.5)	
TIVA (cases, %)	1 (1.6)	3 (6.5)	
**Anesthetics**			
Sevoflurane	43 (69.3)	16 (34.8)	<0.01 *
**Operating**			
Ope time (min), Ave ± SD	701.7 ± 105.2	688.1 ± 109.2	0.51
Bleeding volume (g), Mean ± SD	517.1 ± 324.7	496.2 ± 250.9	0.74
Intraoperative blood transfusion (cases, %)	11 (17.7	5 (10.9)	0.41
**Flap**			0.49
ALT (cases, %)	25 (40.3)	18 (39.1)	
RAMF (cases, %)	17 (27.4)	17 (37.0)	
Vascularised fibula flap (cases, %)	20 (32.3)	11 (23.9)	
**Postoperative complications**			
EPOP (cases, %)	13 (21.0)	19 (41.3)	0.02 *
EPOD (cases, %)	15 (24.2)	14 (30.4)	0.51

**Table 2:** Subgroup analysis of SG and NSG group (* *p* < 0.05).

Variable	SG (N= 62)	NSG (N = 36)	*p*-Value
**Patient background**			
male:female	42:20	22:14	0.51
Age, mean ± SD (years)	69.5 ± 9.10	64.8 ± 1.8	0.51
Age, over 75 years (cases, %)	12 (19.4)	8 (22.2)	0.73
BMI, Ave ± SD	22.2 ± 2.96	21.4 ± 3.60	0.09
Perfomance Status (0–4), Ave ± SD	0.03 ± 0.06	0.2 ± 0.40	0.02 *
ASA, Ave ± SD	2.34 ± 0.58	2.4 ± 0.48	0.86
Hypertension (cases, %)	31 (50.0)	14 (38.9)	0.29
**Preoperative condition**			
Preoperative Alb (g/dL), Ave ± SD	4.0 ± 0.32	3.98 ± 0.45	0.7
Preoperative Cre(mg/dL), Ave ± SD	0.71 ± 0.38	0.70 ± 0.17	0.18
Preoperative Hb (g/dL), Ave ± SD	13.0 ± 1.53	13.1 ± 0.30	0.45
**Anesthesia method**			0.27
Inhalation anesthesia (cases, %)	61 (98.4)	34 (94.4)	
TIVA (cases, %)	1 (1.6)	2 (5.6)	
**Anesthetics**			0.02 *
Sevoflurane (cases, %)	43 (69.4)	16 (44.4)	
**Operating**			
Ope time (min) Ave ± SD	686.5 ± 86.0	693.6 ± 114.0	0.72
Bleeding volume (g) Ave ± SD	416.5 ± 261.8	525.4 ± 261.1	0.9
Intraoperative blood transfusion (cases, %)	11 (17.7)	4 (11.1)	0.38
**Postoperative management**			
Postoparative opioid (cases, %)	39 (62.9)	19 (52.8)	0.4
Postoperative NRS, Ave ± SD	0.15 ± 0.27	0.78 ± 1.93	0.02 *
Postoperative Na (mEq/L), Ave ± SD	140.5 ± 2.18	140.0 ± 3.35	0.8
Postoperative CRP (mg/dL), Ave ± SD	8.0 ± 2.2	9.19 ± 2.53	0.07
**Postoperative complications**			
EPOP (cases, %)	13 (21.0)	14 (38.9)	0.06
EPOD (cases, %)	15 (24.2)	8 (22.2)	0.82

**Table 3:** Subgroup analysis of the RSG and NSG (* *p* < 0.05).

Variable	RSG (N = 10)	NSG (N = 36)	*p*-Value
**Patient background**			
male:female	9:1	22:14	0.08
Age, Ave ± SD (years)	70.4 ± 7.47	64.8 ± 1.8	0.16
Age, over 75 years (cases, %)	4 (40.0)	8 (22.2)	0.25
BMI, Ave ± SD	24.5 ± 3.5	21.4 ± 3.60	0.03 *
Perfomance Status (0–4), Ave ± SD	0.1 ± 0.31	0.2 ± 0.40	0.46
ASA, Ave ± SD	2.4 ± 0.52	2.4 ± 0.48	0.84
Hypertension (cases, %)	8 (80.0)	14 (38.9)	0.02 *
**Preoperative condition**			
Preoperative Alb (g/dL), Ave ± SD	3.83 ± 0.43	3.98 ± 0.45	0.37
Preoperative Cre(mg/dL), Ave ± SD	0.93 ± 0.29	0.70 ± 0.17	0.02
Preoperative Hb (g/dL), Ave ± SD	13.4 ± 2.12	13.1 ± 1.33	0.55
**Anesthesia method**			0.61
Inhalation anesthesia (cases, %)	9 (90.0)	34 (94.4)	
TIVA (cases, %)	1 (10.0)	2 (5.6)	
**Anesthetics**			
Sevoflurane (cases, %)	0 (0)	16 (44.4)	0.01 *
**Operating**			
Ope time (min) Ave ± SD	668.5 ± 92.3	693.6 ± 114.0	0.52
Bleeding volume (g) mean ± SD	391 ± 184	525.4 ± 261.1	0.15
Intraoperative blood transfusion (cases, %)	1 (10)	4 (11.1)	0.92
**Postoperative management**			
Postoparative opioid (cases, %)	6 (60.0)	19 (52.8)	0.69
Postoperative NRS, Ave ± SD	0.40 ± 0.84	0.78 ± 1.93	0.5
Postoperative Na (mEq/L), Ave ± SD	140.1 ± 1.79	140.0 ± 2.75	0.94
Postoperative CRP (mg/dL), Ave ± SD	7.72 ± 1.61	9.19 ± 2.53	0.19
**Postoperative complications**			
EPOP (cases, %)	5 (50.0)	14 (38.9)	0.52
EPOD (cases, %)	6 (60.0)	8 (22.2)	0.02 *

**Table 4:** Subgroup analysis of the SG and RSG (* *p* < 0.05).

Variable	SG (N = 62)	RSG (N = 10)	*p*-Value
**Patient background**			
Gender, male:female	42:20	9:1	0.15
Age, Ave ± SD	66.3 ± 11.0	70.4 ± 7.47	0.24
Age, over 75 years (cases, %)	12 (19.4)	4 (40.0)	0.14
BMI, mean ± SD	22.7 ± 3.63	24.5 ± 3.5	0.15
Perfomance Status (0–4), Ave ± SD	0.03 ± 0.18	0.1 ± 0.31	0.38
ASA, Ave ± SD	2.34 ± 0.65	2.4 ± 0.52	0.77
Hypertension (cases, %)	31 (50.0)	8 (80.0)	0.07
**Preoperative condition**			
preoperative Alb (g/dL) Ave ± SD	3.94 ± 0.42	3.83 ± 0.43	0.43
preoperative Cre (mg/dL), Ave ± SD	0.91 ± 1.28	0.93 ± 0.29	0.97
preoperative Hb (g/dL) Ave ± SD	12.8 ± 0.23	13.4 ± 2.12	0.37
**Anesthesia method**			0.13
Inhalation anesthesia (cases, %)	61 (98.4)	9 (90.0)	
TIVA (cases, %)	1 (1.6)	1 (10.0)	
**Anesthetics**			
Sevoflurane (cases, %)	43 (69.4)	0 (0)	<0.01 *
**Operating**			
Ope time (min), Ave ± SD	701.7 ± 105.2	668.5 ± 92.3	0.33
Bleeding volume (g), Mean ± SD	517.1 ± 324.7	391 ± 184	0.2
Intraoperative blood transfusion (cases, %)	11 (17.7)	1 (10)	0.34
**Postoperative management**			
Postoparative using opioid (cases, %)	39 (62.9)	6 (60.0)	0.86
Postoperative NRS, Ave ± SD	0.15 ± 0.67	0.40 ± 0.84	0.34
Postoperative Na (mEq/L), Ave ± SD	140.2 ± 2.80	140.1 ± 1.79	0.93
Postoperative CRP (mg/dL), Ave ± SD	8.0 ± 2.9	7.72 ± 1.61	0.78
**Postoperative complications**			
EPOP (cases, %)	13 (21.0)	5 (50.0)	0.04 *
EPOD (cases, %)	15 (24.2)	6 (60.0)	0.02 *

**Table 5:** Examination of EPOP (* *p* < 0.05).

Variable	EPOP Group (N = 32)	No EPOP Group (N = 76)	*p*-Value
**Patient background**			
Gender, male:female	26:6	47:29	0.07
Age, mean ± SD (years)	71 ± 9.7	67 ± 8.7	0.71
Age, over 75 years (cases, %)	8 (25.0)	16 (21.1)	0.65
BMI (ave ± SD)	23.0 ± 3.36	22.3 ± 2.73	0.44
Perfomance Status (0–4), Ave ± SD	0.09 ± 0.16	0.09 ± 0	0.97
ASA, Ave ± SD	2.19 ± 2	2.42 ± 2	0.06
History of respiratory disease (cases, %)	5 (15.6)	6 (7.9)	0.24
Smoking habit (cases, %)	20 (62.5)	41 (53.9)	0.41
Hypertension (cases, %)	15 (46.9)	38 (50.0)	0.77
**Preoperative condition**			
Preoperative Alb (g/dL), Ave ± SD	3.8 ± 0.46	4.0 ± 0.28	0.03 *
Preoperative Hb (g/dL), Ave ± SD	13.1 ± 1.68	13.2 ± 1.22	0.85
Preoperative Cre (mg/dL), Ave ± SD	0.75 ± 0.15	0.71 ± 0.33	0.68
Cancer stage	3.78 ± 0.38	3.75 ± 0.39	0.17
**Anesthesia method**			0.83
Inhalation anesthesia (cases, %)	31 (96.9)	73 (96.1)	
TIVA (cases, %)	1 (3.1)	3 (3.9)	
**Anesthetics**			
Sevoflurane (cases, %)	18 (56.3)	41 (54.0)	0.83
**Operating**			
Intraoperative blood transfusion (cases, %)	6 (18.8)	10 (13.2)	0.55
Ope time (min)	699.7 ± 84.6	695.2 ± 87.2	0.46
Bleeding volume (g)	400 ± 240	501.5 ± 219.4	0.71
**Postoperative management**			
Postoperative Na (mEq/L), Ave ± SD	140.7 ± 1.78	139.9 ± 2.17	0.28
Postoperative CRP (mg/dL), Ave ± SD	8.85 ± 2.43	8.16 ± 2.10	0.13
Postoperative opioid (cases, %)	18 (56.3)	46 (61.0)	0.68
Sedation (cases, %)	18 (56.3)	54 (71.1)	0.18
Duration of sedation (h), mean ± SD	10 ± 8.6	11 ± 9.4	0.39
**Complications**			
EPOD (cases, %)	13 (41.0)	16 (21.1)	0.03 *

**Table 6:** Logistics regression analysis of EPOP (* *p* < 0.05).

	B	Wald	*p*-Value	95% CI
Preoperative Alb	−1.183	4.66	0.03 *	−2.32–0.158
Sedation	0.522	5.55	0.06	−0.410–1.447

**Table 7:** Examination of EPOD (* *p* < 0.05).

Variable	EPOD Group (N = 29)	No EPOD Group (N = 79)	*p*-Value
**Patient background**			
Gender, male:female	22:7	51:28	0.35
Age, mean ± SD (years)	71 ± 7.08	65 ± 9.25	0.05
Age, over 75 years (cases, %)	11 (38.0)	13 (26.4)	0.02 *
BMI, Ave ± SD	22.4 ± 3.66	22.5 ± 2.66	0.96
Perfomance Status (0–4), Ave ± SD	0.20 ± 0.31	0.05 ± 0.09	0.02 *
ASA, Ave ± SD	2.51 ± 0.54	2.29 ± 0.49	0.08
Hypertension (cases, %)	18 (62.1)	35 (44.3)	0.1
**Preoperative management**			
Preoperative Alb (g/dL), Ave ± SD	3.77 ± 0.37	4.0 ± 0.30	0.02 *
Preoperative Cre(mg/dL), Ave ± SD	0.74 ± 0.16	0.71 ± 0.31	0.85
Preoperative Hb (g/dL), Ave ± SD	13.2 ± 1.67	13.1 ± 1.24	0.52
**Anesthesia method**			0.93
Inhalation anesthesia (cases, %)	28 (96.6)	76 (96.2)	
TIVA (cases, %)	1 (3.4)	3 (3.8)	
**Anesthetics**			
Sevoflurane (cases, %)	13 (44.8)	46 (58.2)	0.28
**Operating**			
Intraoperative blood transfusion (cases, %)	3 (10.3)	13 (16.5)	0.55
Ope time (min) Ave ± SD	703.0 ± 81.0	693.3 ± 87.3	0.67
Bleeding volume (g) Ave ± SD	482.2 ± 190.1	517.7 ± 238.4	0.57
**Postoperative management**			
Postoperative Na (mEq/L), Ave ± SD	139.7 ± 2.48	140.3 ± 1.91	0.27
Postoperative CRP (mg/dL), Ave ± SD	7.56 ± 1.88	8.65 ± 2.36	0.1
Postoperative NRS, Ave ± SD	0.38 ± 0.65	0.38 ± 0.68	0.99
Postoperative opioid (cases, %)	17 (58.6)	47 (56.5)	0.93
Sedation (cases, %)	21 (72.4)	51 (64.6)	0.49
Duration of sedation (h), mean ± SD	13 ± 10.6	9 ± 8.6	0.07
**Postoperative complications**			
EPOP (cases, %)	13 (44.8)	19 (24.1)	0.04 *

## Data Availability

All authors share the research data.
